# Identification of LDHA as a Potential Therapeutic Target for Pulmonary Hypertension Through Modulation of Endothelial‐To‐Mesenchymal Transition

**DOI:** 10.1111/jcmm.70692

**Published:** 2025-07-08

**Authors:** Maozhong Yao, Keyan Zhong, Xinbin Zheng, Zhaoxin Yang, Chunying Li, Yong Gu, Zhanjuan Chen

**Affiliations:** ^1^ Clinical Research Center Affiliated Chinese Medicine Hospital of Hainan Medical University (Hainan Academy of Medical Sciences) Haikou Hainan China; ^2^ Clinical Research Center, Hainan Hospital, Guangdong Provincial Hospital of Chinese Medicine Guangzhou University of Chinese Medicine Haikou Hainan China; ^3^ Hainan Clinical Center for Encephalopathy of Chinese Medicine Haikou Hainan China; ^4^ Hainan Clinical Research Center for Preventive Treatment of Diseases Haikou Hainan China; ^5^ Clinical Skills Experimental Teaching Center Hainan Medical University (Hainan Academy of Medical Sciences) Haikou Hainan China; ^6^ Research Center for Drug Safety Evaluation of Hainan Province Hainan Medical University (Hainan Academy of Medical Sciences) Haikou Hainan China; ^7^ Department of Pulmonary Diseases Affiliated Chinese Medicine Hospital of Hainan Medical University (Hainan Academy of Medical Sciences) Haikou Hainan China; ^8^ Department of Pulmonary Diseases, Hainan Hospital, Guangdong Provincial Hospital of Chinese Medicine Guangzhou University of Chinese Medicine Haikou Hainan China; ^9^ School of Pharmacy Hainan Medical University (Hainan Academy of Medical Sciences) Haikou Hainan China

**Keywords:** bioinformatics analysis, endothelial‐to‐mesenchymal transition, FX11, lactate dehydrogenase A, pulmonary hypertension, therapeutic target

## Abstract

Endothelial‐to‐mesenchymal transition (EndMT) induced by dysfunctional pulmonary artery endothelial cells (PAECs) is regarded as an initiating and pivotal factor in pulmonary hypertension (PH). This study focuses on identifying a novel therapeutic target for regulating EndMT in PH. A comprehensive analysis of 2 hypoxic PAECs datasets yielded 310 overlapping upregulated and 229 downregulated differentially expressed genes (DEGs). These upregulated DEGs were primarily enriched in HIF‐1 signalling pathway and glycolysis/gluconeogenesis, while downregulated only in spliceosome, as indicated by KEGG. Through PPI network analysis and the application of MCC algorithms, 5 hub genes were identified among these upregulated DEGs: GAPDH, LDHA, ALDOA, PFKL, and PFKP. Their enrichment in the 2 aforementioned pathways was confirmed by cross‐pathway DEGs analysis and ClueGo. Among the hub genes, LDHA was chosen as the key gene based upon expression and correlation analysis of the validation set from PH patients. Subsequent GSEA also revealed the enrichment of LDHA in these 2 pathways. Additionally, the increased expression of LDHA protein in tissues and cells was confirmed, and the elevated enzymatic activity of LDHA in clinical serum samples was also verified. From 2 online databases, 4 LDHA inhibitors were filtered out, and the stable binding between the inhibitors and the LDHA protein was confirmed through molecular docking and molecular dynamics simulation. Finally, the experimental results indicated that one of the inhibitors FX11 reversed EndMT by inhibiting the lactate‐SNAI1 axis, thereby alleviating hypoxia‐induced PH. The potential of LDHA as a therapeutic target for PH by modulating EndMT was proposed in this study.

## 1 Introduction

Pulmonary hypertension (PH) is a life‐threatening disease characterised primarily by vascular remodelling, which leads to increased strain on the right ventricle and may ultimately result in heart failure or mortality [1]. Although significant progress has been made in understanding the pathogenesis, diagnosis, pharmacotherapy, and treatment modalities of PH over the past 30 years, the 1‐year mortality rate after confirmed diagnosis remains at 14.5%, with a 5‐year survival rate of only 53.6% [2]. Among the various treatment modalities, medications continue to be a convenient, accessible, and cost‐effective solution. Currently, available medications for PH target three primary pathways involved in PH: stimulating the nitric oxide‐cyclic guanosine monophosphate biological pathway, enhancing prostacyclin effects on receptors, and blocking the endothelin pathway [3]. These drugs primarily aim at improving vasodilatory properties and enhancing cardiopulmonary function. However, they still face significant limitations due to their inability to halt or reverse the progression of PH [4].

Pulmonary arterial remodelling, a crucial element of PH, encompasses several pathological mechanisms, including aberrant proliferation of pulmonary artery endothelial cells (PAECs) and smooth muscle cells (PASMCs), extracellular matrix deposition, and endothelial‐to‐mesenchymal transition (EndMT) [5]. EndMT is a complex biological process where endothelial cells undergo a phenotypic transition towards an interstitial state, marked by the downregulation of vascular endothelial cell markers such as cadherin 5 (CDH5, also known as VE‐cadherin) and platelet and endothelial cell adhesion molecule 1 (PECAM1, also called CD31) and the upregulation of mesenchymal cell markers like vimentin (VIM) and actin alpha 2, smooth muscle (ACTA2, also known as α‐SMA) [6]. Previous research has identified several molecules, including transforming growth factor β1 receptor 1 [7], inositol 1,4,5‐trisphosphate receptor 3 [8], and bone morphogenetic protein [9] as key regulators involved in EndMT. More recently, our research and others have highlighted the significance of EndMT as a novel contributor to the pathogenesis of PH [10, 11]. Consequently, targeting EndMT as a therapeutic approach offers significant potential for preventing or reversing the progression of PH [12]. Several substances, including aspirin [13], eNAMPT‐neutralising mAb [14], and naringin [15], have been evaluated in animal models for their ability to suppress EndMT and treat PH. Thus, identifying therapeutic targets in genes regulating EndMT is a promising direction for the development of new anti‐PH drugs.

In this study, we employed R, along with the online databases STRING and Cytoscape, to pinpoint a key gene involved in hypoxia‐induced EndMT from transcriptome datasets of hypoxic human pulmonary artery endothelial cells (HPAECs) and lung tissues from patients with PH. To confirm the therapeutic target role of this key gene in EndMT, we screened for compounds targeting the key gene using online databases BindingDB and ZINC20. Subsequently, we validated the binding affinity of these compounds to the key gene using molecular docking and molecular dynamics simulation (MDS) techniques. Ultimately, we demonstrated the role of one compound in mitigating hypoxia‐induced PH by inhibiting EndMT and elucidated the underlying molecular mechanism. The detailed workflow employed in this study was illustrated in Figure S1.

## 2 Materials and Methods

### 2.1 Data Sources

The transcriptional data related to EndMT in HPAECs were obtained from datasets GSE163827 and GSE157231, which were downloaded from the Gene Expression Omnibus (GEO) database (https://www.ncbi.nlm.nih.gov/). GSE163827 was generated using platform GPL18573 and consisted of 9 samples of HPAECs, including 3 hypoxia‐treated samples and 3 normoxia control samples selected specifically for this study. Meanwhile, GSE157231 used platform GPL11154 and comprised 24 samples of HPAECs, with 4 hypoxia‐treated samples and 4 normoxia control samples chosen for further research. Another gene expression profile, GSE113439, was selected to validate the hub genes. This dataset included lung tissue samples from 11 normal individuals and 15 PH patients.

### 2.2 Identification of Overlapping Differentially Expressed Genes

The R package DESeq2 [16] was employed to analyse the gene expression of GSE157231 and GSE163827 and identify the differentially expressed genes (DEGs) with an adjusted *p*‐value < 0.05 and a criterion of |log2(FC)| > 0.26. Volcano plots and heatmaps depicting the DEGs were generated using R software. Additionally, the overlapping DEGs between the 2 datasets were determined through the R package VennDiagram [17].

### 2.3 Functional Enrichment Analysis

The gene ontology (GO) and the Kyoto Encyclopedia of Genes and Genomes (KEGG) analyses were used to elucidate the potential functions of the DEGs using the R clusterProfiler package [18]. Categories such as cellular component (CC), biological process (BP), and molecular function (MF) were all included in the GO analysis.

### 2.4 Protein–Protein Interaction Network and Module Analyses

The protein–protein interaction (PPI) network of the DEGs was constructed with database STRING [19]. To visualise and analyse the PPI network, Cytoscape software [20] was used. The PPI network was then analysed by MCODE [21] and CytoHubba [22] plugins in Cytoscape. Based on the maximum clique centrality (MCC) algorithm [23] in CytoHubba, the hub genes were identified and ranked. The GO and KEGG enrichment analysis of the hub genes was conducted using the ClueGo plug‐in.

### 2.5 Data Verification

The RNA expression dataset GSE113439 from clinical samples was utilised as the validation set to verify the hub genes. The expression of marker genes of EndMT and the hub genes were analysed by *t* test in R. Moreover, the correlation between the marker genes and hub genes was explored using the R packages corrplot and ggstatsplot.

### 2.6 Gene Set Enrichment Analysis

Gene set enrichment analysis (GSEA) was conducted to investigate the signalling pathway in which the key gene might be involved. An adjusted *p*‐value < 0.05 was considered statistically significant.

### 2.7 Cell Culture and Treatment

HPAECs (Shxybio, China) were cultured in Dulbecco's Modified Eagle Medium containing 10% fetal bovine serum (ExCell, China) at 37°C and 5% CO_2_. Prior to hypoxia treatment, all cells were synchronised with 1% serum for 12 h. Then the cells were subjected to normoxia (21% O_2_) or hypoxia (1% O_2_), either with or without FX11 (5 μM, MCE, USA), AZ33 (20 μM, MCE, USA), and lactate (50 mM, MCE, USA) for 48 h. In addition to hypoxia, DMSO (1‰, MCE, USA) was added as a solvent control.

### 2.8 Clinical Sample Testing

LDH enzyme activity and lactate concentration of patients with PH were obtained from their medical records. Data from healthy controls were obtained from serum samples collected after routine physical examinations. All data were measured using a fully automated biochemical analyser. This study was approved by the Ethics Committee of Hainan Hospital, Guangdong Provincial Hospital of Chinese Medicine, China, with the requirement for informed consent waived (Approval Number: HNSZYY‐2024‐LL‐071; Approval Date: July 24, 2024).

### 2.9 Protocols for Animal Experiment

Male C57BL/6J mice (8 weeks old) were obtained from Guangdong Medical Laboratory Animal Center, China (SCXX (YUE) 2022–0002). All mice received humane care in accordance with the Guide for the Care and Use of Laboratory Animals (2011 edition) and were divided randomly into 4 groups: the control group was exposed to normoxia (21% O_2_) for 3 weeks; the hypoxia group was placed in a hypoxic chamber (10% O_2_) for 3 weeks; the DMSO group was treated with DMSO (MCE, USA) diluted in saline under the same hypoxic conditions for 3 weeks (20 mL/kg/day); and the FX11 (MCE, USA) group was injected intraperitoneally with FX11 under hypoxic conditions for 3 weeks (2 μg/kg/day, 10% O_2_). Additionally, the latter 3 groups of mice were subcutaneously injected with SU5416 (20 mg/kg, once weekly for 3 weeks, MCE, USA). The study protocol was approved by the Institutional Animal Care and Use Committee of Hainan Hospital, Guangdong Provincial Hospital of Chinese Medicine (Approval Number: IACUC‐HHGPHCM‐2404002; Approval Date: April 30, 2024).

### 2.10 Measurement of RVSP by Right Heart Puncture

After the feeding period, right heart puncture method was used to measure right ventricular systolic pressure (RVSP). The procedure was as follows: after anaesthetising the mice, tracheal intubation was performed. The chest was then opened along the midline of the sternum, and a size 7 needle filled with heparinized saline was inserted into the right ventricle. The other end of the needle was connected to a catheter attached to a pressure transducer to record pressure changes through a biological function experimental system.

### 2.11 Immunofluorescence Staining

After pretreatment, the lung tissue slices or cells were incubated with primary antibodies against LDHA (1:100, Abclonal, China), ACTA2 (1:100, Sigma‐Aldrich, USA), PECAM1 (1:100, Abclonal, China), SNAI1 (1:100, Abclonal, China) at 4°C overnight. Subsequently, they were incubated with the secondary fluorescent antibody and DAPI at room temperature. Finally, the slices or cells were observed and imaged under a fluorescence microscope.

### 2.12 Western Blot

Samples of HAPECs were lysed. The protein concentration in the lysates was measured. Equal amounts of total proteins from different groups were separated by 10% SDS‐PAGE gel and then transferred to PVDF membranes (Millipore, USA). The membranes were incubated with primary antibodies against LDHA (1:1000, CST, USA), CDH5 (1:1000, Abclonal, China), VIM (1:1000, CST, USA), SNAI1 (1:1000, Abclonal, China), and GAPDH (1:1000, Proteintech, China) overnight at 4°C, respectively. After incubation with HRP‐labelled secondary antibody at room temperature for 1 h, the proteins were detected and quantified using an image analysis system.

### 2.13 Screening of the Targeted Compounds

Compounds targeting the key gene were separately searched in the BindingDB and ZINC20 databases. The substances obtained from ZINC20 had overlap with those from BindingDB.

### 2.14 Molecular Docking Analysis

The LDHA protein structure (PDB code: 4OJN) from the Protein Data Bank was processed using Pymol software (version 2.6.0a0). The structure data files of compounds were acquired from PubChem. To elucidate the specific binding mode between the protein and compounds, AutoDock Vina [24] was employed for docking simulations, resulting in the generation of complex structures.

### 2.15 Molecular Dynamics Simulation

The MDSs of the 2 complexes were conducted using GROMACS 2020.1 [25], employing the CHARMM36‐Jul2020 force field. The complex was solvated in TIP3P water and immersed in a dodecahedron box with a solvent margin of at least 1 nm on all sides. Thereafter, the system was neutralised by the addition of Na^+^ and Cl^−^ ions, and a 0.15 M NaCl solution was introduced. Energy minimization was performed using the steepest descent algorithm until the maximum force reached below 1000 kJ/mol/nm. Subsequently, it was equilibrated for 100 ps. Finally, MDSs of the complexs were conducted for 100 ns.

### 2.16 Morphological Observation

The cells were observed and photographed in bright‐field microscopy.

### 2.17 LDH Enzyme Activity Testing in Cells and Tissues

The lactate dehydrogenase (LDH) enzyme activity assay was conducted according to the instructions provided in the kit (NJJCBIO, China). Lysed samples and reagents were added to each well. The absorbance was measured at 450 nm, followed by the calculation of LDH activity using a designated formula.

### 2.18 Lactate Concentration Measurement in Tissues

In accordance with the instructions supplied in the kit (NJJCBIO, China), the lactate concentration in animal lung tissue was measured. The homogenate, enzyme working solution, chromogenic reagent, and stop solution were sequentially added to the corresponding well. The absorbance of the mixture was measured at 530 nm.

### 2.19 Haematoxylin and Eosin Staining

The tissues were pretreated and sectioned at a thickness of 5 μm. The sections were stained with H&E and observed under a microscope to visualise the morphology.

### 2.20 Statistical Analysis

Differential analysis of the sequencing and microarray data was conducted using R. The data such as WB, enzyme activity, and lactate concentration were analysed with SPSS software (Version 26.0) and the results were expressed as mean ± S.E.M. (stand error of mean). Statistical difference of experimental data was determined by independent‐sample *t*‐tests for two groups or by one‐way ANOVA followed by Newman–Student–Keuls test for multiple group comparisons. A *p*‐value < 0.05 was considered statistically significant.

## 3 Results

### 3.1 Identification of DEGs

The datasets used in this study were presented in Table 1 and Figure S1. The results of principal component analysis (PCA) conducted on the 2 training sets, GSE163827 and GSE157231, were presented in Figure S2A,B, respectively. A total of 3850 DEGs were identified from GSE163827, with 1930 upregulated and 1920 downregulated genes (Figure S2C,E). Additionally, 1026 DEGs were selected from GSE157231, comprising 581 upregulated and 445 downregulated genes (Figure S2D,F).

**TABLE 1:** The GEO datasets used in this study.

ID	Platform	Sample type	Data type	Sample number	Sample used in this study	Control used in this study	Year
GSE163827	GPL18573	HPAECs	Expression profiling by high throughput sequencing	18	6	3	2020
GSE157231	GPL11154	HPAECs	Expression profiling by high throughput sequencing	24	8	4	2020
GSE113439	GPL6244	Human lung	Expression profiling by array	26	26	11	2018

### 3.2 Functional Enrichment Analyses of the Overlapping Upregulated DEGs

**FIGURE 1:**
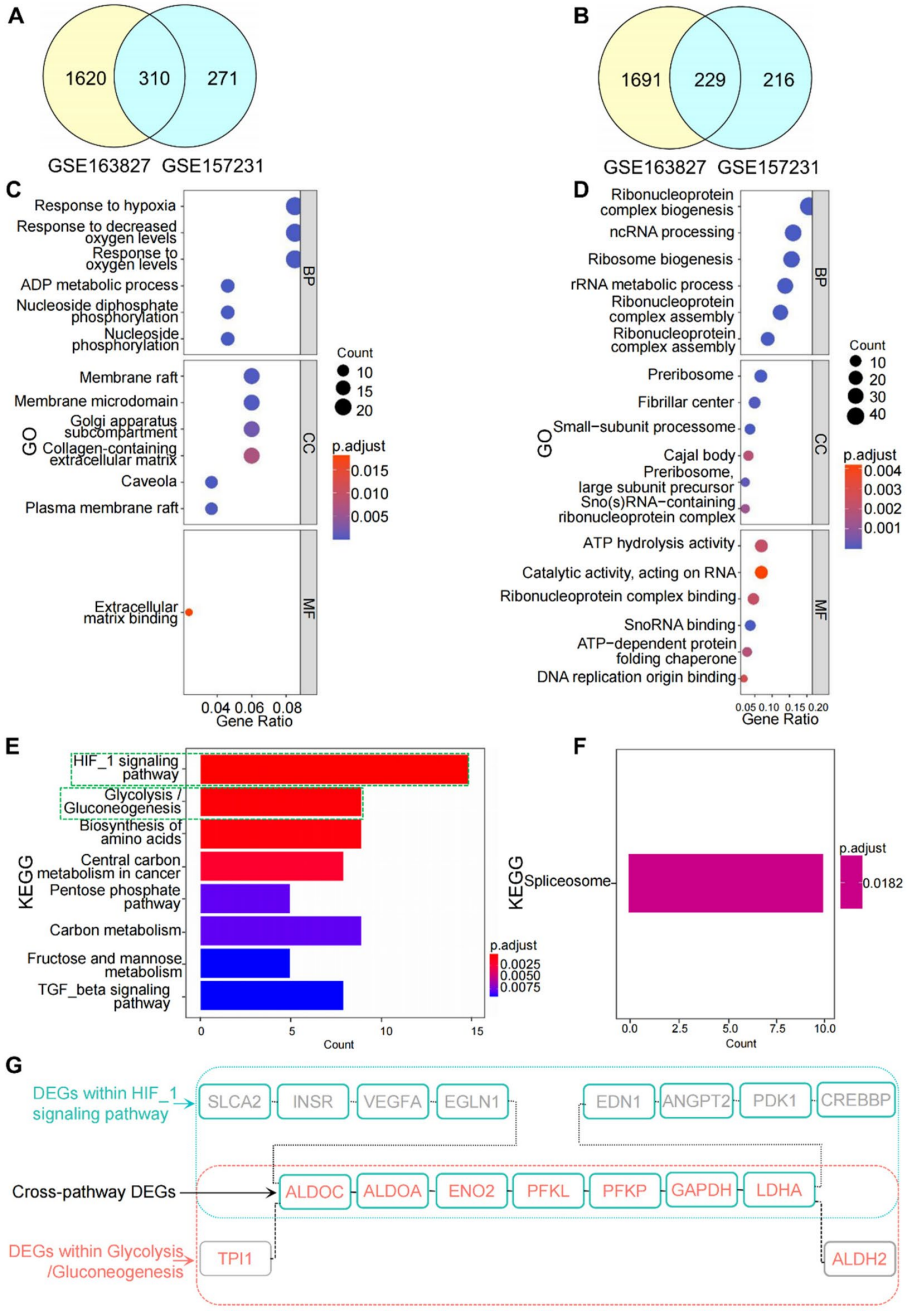
GO and KEGG enrichment analysis for overlapping DEGs. Venn diagram showing overlapping DEGs from the 2 datasets for upregulated (A) and downregulated (B) genes. GO analysis for upregulated (C) and downregulated (D) DEGs. KEGG analysis for upregulated (E) and downregulated (F) DEGs. (G) The cross‐pathway DEGs between the HIF‐1 signalling pathways and glycolysis/gluconeogenesis.

We discovered 310 overlapping upregulated DEGs between the 2 datasets (Figure 1A). Subsequently, these overlapping DEGs were subjected to GO analysis. For CC, they were primarily enriched in membrane raft, membrane microdomain, and collagen‐containing extracellular matrix; regarding BP, significant enrichment was observed for terms related to hypoxia, such as response to hypoxia, response to decreased oxygen levels, and response to oxygen levels; the MF terms associated with these DEGs included extracellular matrix binding (Figure 1C). Moreover, KEGG analysis indicated that the DEGs were mainly enriched in HIF‐1 signalling pathway and glycolysis/gluconeogenesis (Figure 1E). These DEGs, specifically ALDOC, ALDOA, ENO2, PFKL, PFKP, GAPDH, and LDHA, were found to be simultaneously present in the 2 enriched signalling pathways (Figure 1G) and were referred to as “cross‐pathway DEGs”.

As 229 overlapping downregulated DEGs were enriched in non‐specific terms with little relevance to PH (as shown in Figure 1B,D,F), no further research has been conducted on them.

### 3.3 PPI Network and Hub Genes Analyses

**FIGURE 2:**
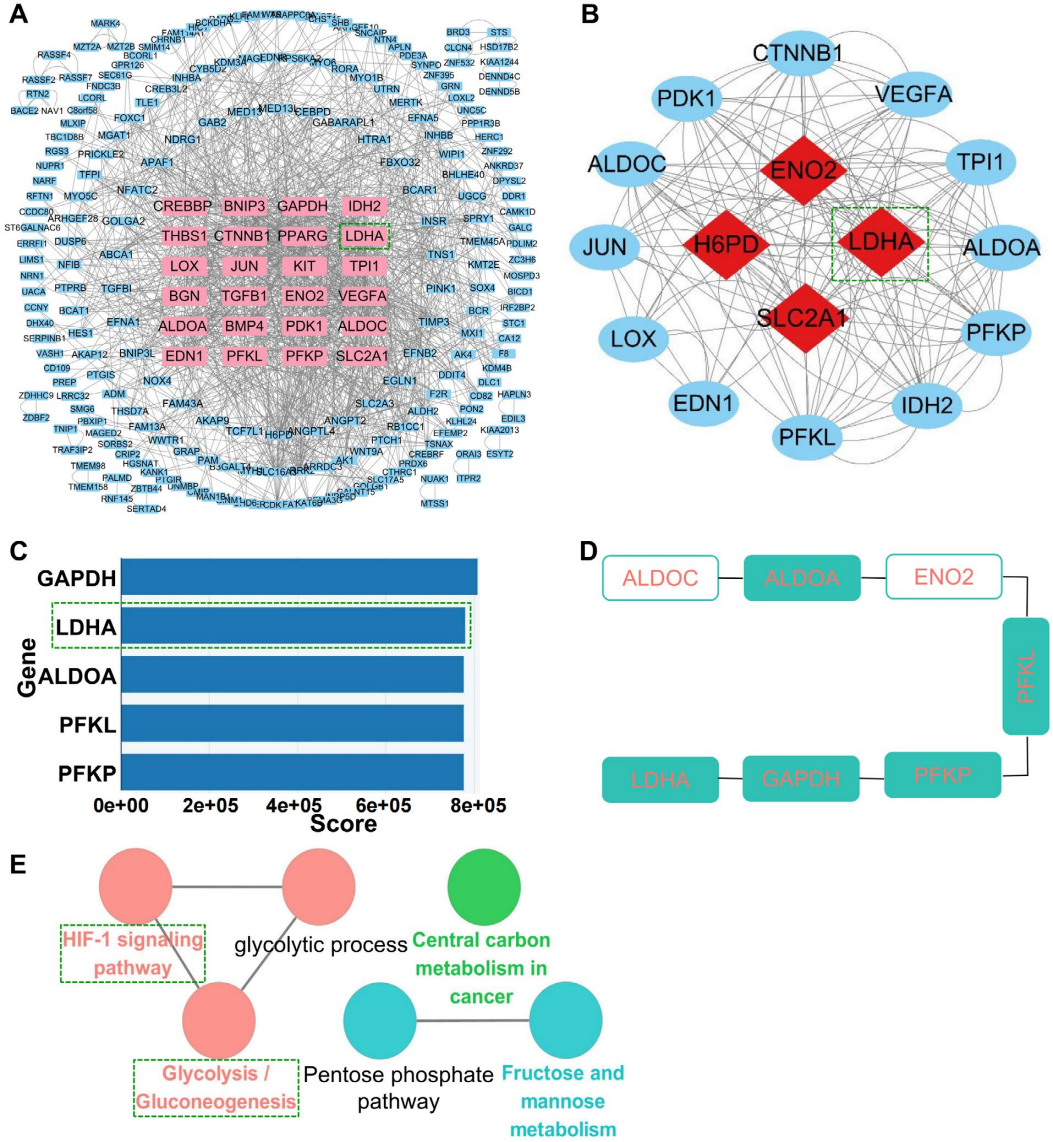
Construction of PPI network for upregulated DEGs and identification of hub genes. (A) PPI network. (B) The module with the highest score. (C) The ladder graph of hub genes. (D) The hub genes within the cross‐pathway DEGs. (E) The ClueGo analysis for hub genes.

We employed the STRING database to construct the PPI network for the overlapping upregulated DEGs. As shown in Figure 2A, the PPI network comprised 241 nodes and 1252 edges, revealing that genes such as IDH2, PFKL, and LDHA occupied core positions. To pinpoint highly interconnected clusters within the network, the MCODE plug‐in in Cytoscape was used, revealing a module with the highest score of 9.6. This module consisted of 16 nodes and 144 edges and covered core genes represented by LDHA (Figure 2B). Additionally, the MCC algorithm implemented in the Cytohubba plug‐in in Cytoscape was utilised to identify 5 hub genes. Listed in descending order of score, they were GAPDH, LDHA, ALDOA, PFKL, and PFKP (Figure 2C). Surprisingly, all 5 hub genes were found to be within the cross‐pathway DEGs (Figure 2D). Since the genes that are closely interconnected within the network play a more fundamental role in regulation, we conducted further investigations into the functions of the hub genes using the ClueGO plug‐in. As illustrated in Figure 2E, these genes remained primarily enriched in the HIF‐1 signalling pathway and glycolysis/gluconeogenesis.

### 3.4 Identification of LDHA as a Key Gene in EndMT

**FIGURE 3:**
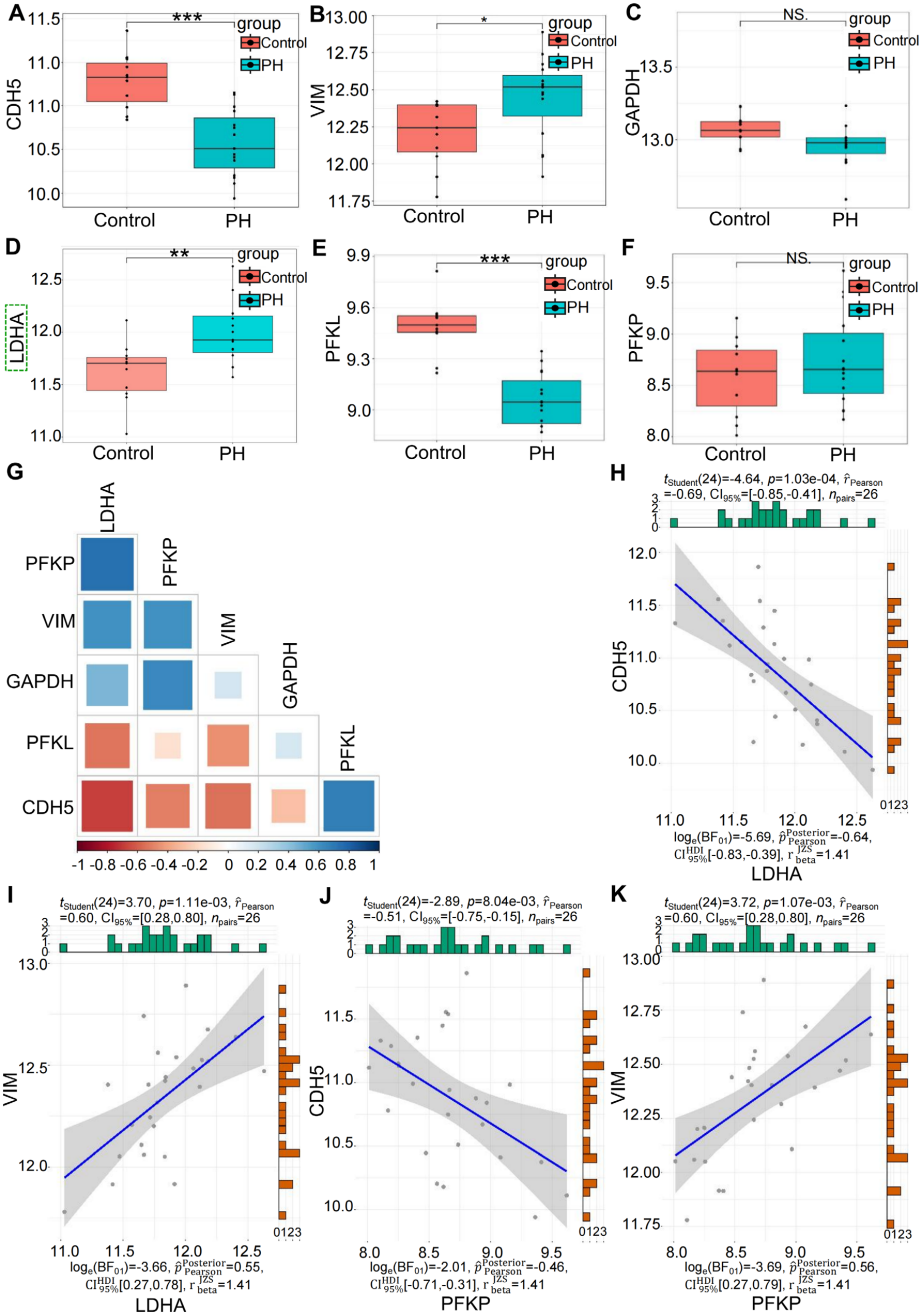
Analysis of marker and hub genes expression and correlation in validation set. Expression of mRNA (A–F). (G) Correlation heatmap. Scatter plots of the correlation between 2 hub genes and 2 marker genes in EndMT (H‐K). *n*_Control_ = 11, *n*_PH_ = 15. NS, not significant; *p < 0.05, **p < 0.01, ***p < 0.001 vs. Control.

To identify the key gene among the hub genes, we analysed the expression of the EndMT marker genes and the 5 hub genes using validation set GSE113439. The results revealed a significant decrease in the expression of the endothelial marker CDH5 (Figure 3A), accompanied by a notable increase in the expression of the mesenchymal marker VIM (Figure 3B). These findings aligned with the anticipated patterns observed in PH, thus verifying the reliability of this dataset as a validation set for EndMT research. Furthermore, the notable upregulation of the hub gene LDHA in the validation set was consistent with the patterns observed in the 2 training sets, as shown in Figure 3D. However, the expression results of the remaining 4 hub genes were inconsistent with the rising trends observed in the training sets. Specifically, there were no significant changes in the expression of GAPDH and PFKP (Figure 3C,F), while PFKL exhibited a significant decrease (Figure 3E). Unfortunately, data for ALDOA was not available. Among the 5 hub genes, LDHA exhibited distinct characteristics: it occupied core positions in the PPI network (Figure 2A), ranked as one of the top 4 members in the module with the highest score (Figure 2B), and was the only one that showed upregulation in all 3 datasets. Therefore, it was initially identified as the key gene.

Next, we analysed the correlations between the hub genes and the marker genes of EndMT using the validation set. The overall correlations were shown in Figure 3G. Among the 4 hub genes with available data, LDHA and PFKP exhibited strong correlations with the 2 marker genes, respectively (Figure 3G–K), suggesting their potential involvement in EndMT. GAPDH displayed weak correlations, whereas PFKL showed a correlation opposite to the trend of EndMT (Figure 3G). Based on the changes in gene expression and its correlation with EndMT marker genes, LDHA has once again been identified as the sole key gene in EndMT.

### 3.5 Integrated in Silico and Experimental Analysis of LDHA

To further explore the functional enrichment of LDHA, GSEA was conducted. In dataset GSE163827, LDHA was found to be enriched in a total of 59 signalling pathways, with Figure S3A displaying 10 representative pathways. Notably, LDHA was again found to be involved in both the HIF‐1 signalling pathway and glycolysis/gluconeogenesis (Figure S3C,E). Similarly, in GSE157231, among the 49 enriched pathways, Figure S3B presented 10 pathways, with LDHA also significantly enriched in the HIF‐1 signalling pathway (Figure S3D). This computational analysis testified that LDHA was still predominantly enriched in the HIF‐1 signalling pathway and glycolysis/gluconeogenesis, further supporting the previous findings.

**FIGURE 4:**
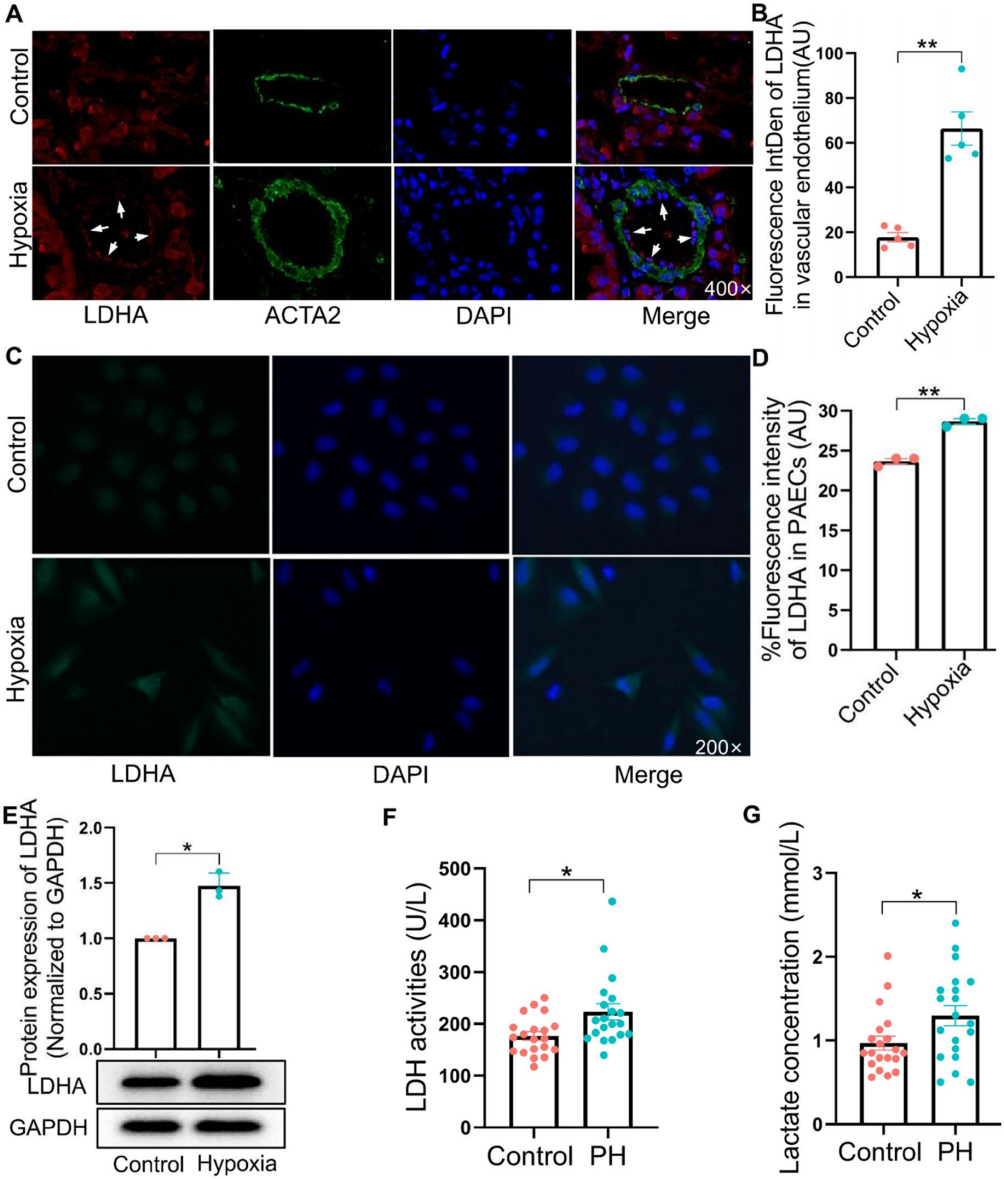
LDHA protein expression and enzyme activity. (A, B) Protein expression in animal vascular endothelium detected by IF (*n* = 5). (C, D) Protein expression in PAECs identified by IF (*n* = 3). (E) Protein expression in PAECs identified by WB (*n* = 3). LDH enzyme activity (F) and lactate concentration (G) in clinical serum samples (*n* = 20). All values are expressed as mean ± S.E.M., *p < 0.05, **p < 0.01 vs. Control.

Given the upregulation of LDHA mRNA expression in the datasets, coupled with documented increase of LDHA protein expression in tumour cells under hypoxic conditions [26], we sought to detect LDHA protein expression in hypoxic pulmonary arteries and PAECs. Immunofluorescence (IF) staining disclosed that exposure to hypoxia led to an upregulation of LDHA expression in hypoxic pulmonary artery endothelium (Figure 4A,B) and cultured HPAECs (Figure 4C–E). These findings confirmed that LDHA protein expression in hypoxic pulmonary artery endothelium and PAECs was indeed elevated under hypoxic conditions, consistent with the trend in the 3 datasets.

Since LDH is composed of LDHA and LDHB subunits, LDHA catalyses the conversion of pyruvate to lactate, while LDHB catalyses the reverse reaction [27]. We used the changes in LDH enzyme activity, along with alterations in lactate concentration, to determine LDHA enzyme activity. The results from human serum analysis indicated that, compared with healthy individuals, both LDH enzyme activity and lactate concentration were significantly increased in PH patients (Figure 4F,G). Furthermore, both LDH enzyme activity and lactate concentration were also significantly increased in lung tissue of PH animal models (Figure 7G,H). These findings revealed an upregulation of LDHA activity in PH patients and animals.

### 3.6 Screening of LDHA Inhibitors

**FIGURE 5:**
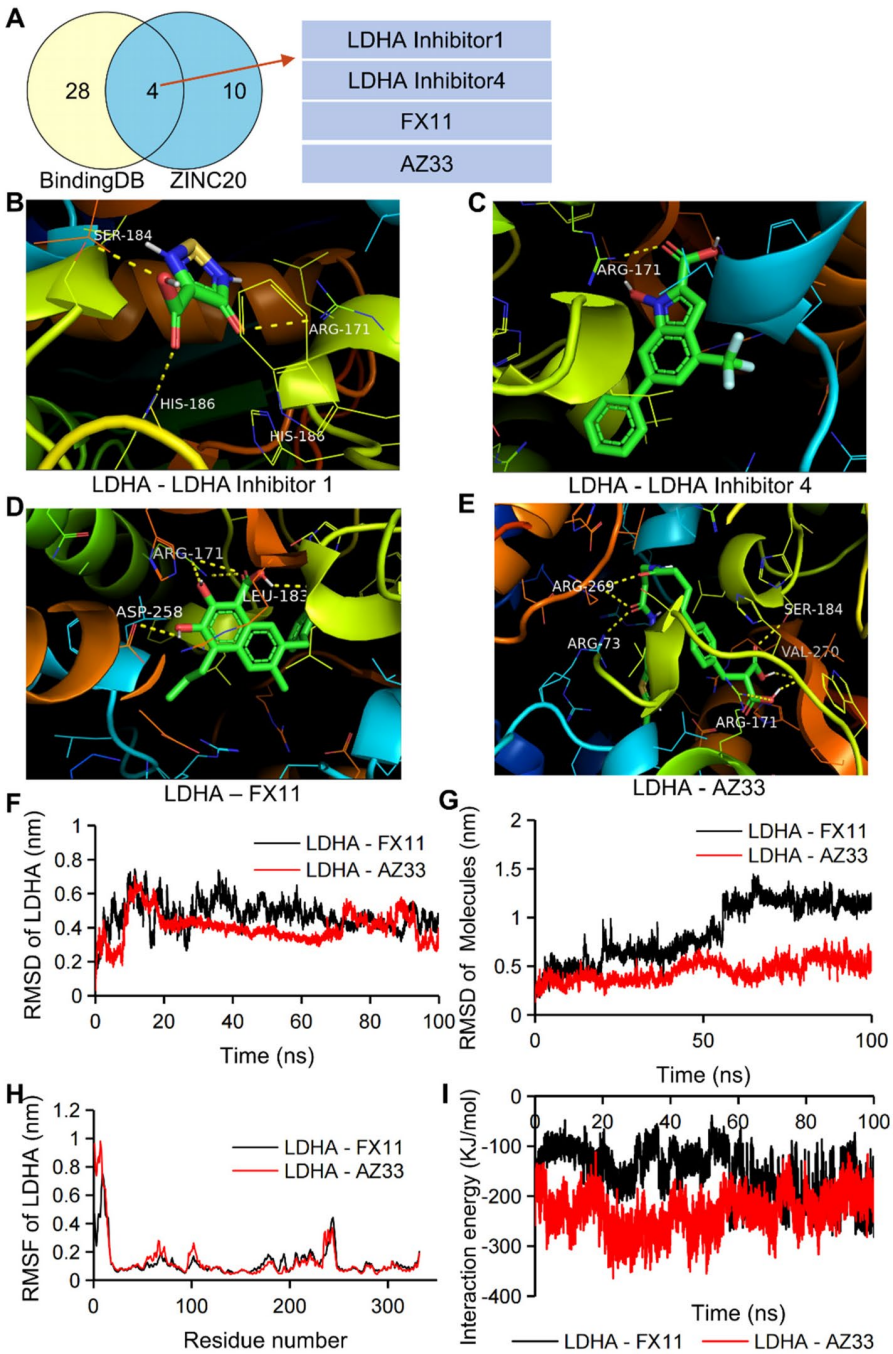
Selection and validation of LDHA inhibitors. (A) 4 overlapping compounds between substances from BindingDB and ZINC20. Molecular docking between LDHA and LDHA Inhibitor 1 (B), LDHA Inhibitor 4 (C), FX11 (D), and AZ33 (E). MDS analysis of complexes formed by LDHA with FX11 or AZ33 (F–I).

To verify the potential of LDHA as a therapeutic target for PH, considering both the significant increase in LDHA protein expression and its role as a catalytic enzyme [28], we conducted a screening of small‐molecule compounds capable of inhibiting LDHA enzyme activity. This screening involved analysing data from the BindingDB and ZINC20 databases. The results indicated that 32 compounds from BindingDB (Table S1) and 14 compounds from ZINC20 (Table S2) have the potential to act as LDHA inhibitors. Subsequently, we filtered out 4 overlapping compounds between the 2 databases using the R package VennDiagram (Figure 5A).

### 3.7 Validation of the Binding Between LDHA Protein and the Filtered Inhibitors

To confirm the reliability of the screened LDHA inhibitor, we used molecular docking to assess the binding potential between LDHA protein and the 4 filtered compounds. The binding site of LDHA with these compounds were shown in Figure 5B–E. Table 2 provided information on binding. Based on Table 2, FX11 and AZ33 were selected as the compounds with potential LDHA inhibitory properties.

**TABLE 2:** The results of LDHA protein docking with 4 overlapping compounds.

No.	InChIKey	Compound name in PubChem	Number of Binding bonds	Binding energy (kcal/mol)
1	FVZITYNLUYJDOE‐UHFFFAOYSA‐N	LDHA Inhibitor 1	6	−5.4
2	KGXDBNBXAPVUIM‐UHFFFAOYSA‐N	LDHA Inhibitor 4	1	−9.0
3	LVPYVYFMCKYFCZ‐UHFFFAOYSA‐N	FX11	5	−8.6
4	SGFJAJFBGVAOFW‐UHFFFAOYSA‐N	AZ33	7	−9.1

MDSs were then performed to validate the stability of the LDHA‐compound complexes. The root mean square deviation (RMSD) analysis revealed that the LDHA protein reached equilibrium after 20 ns (Figure 5F). The RMSD of FX11 stabilised after 60 ns, whereas the RMSD of AZ33 remained stable throughout the entire 100 ns simulation, as depicted in Figure 5G. The root mean square fluctuation (RMSF) analysis showed similar results for LDHA in the presence of both FX11 and AZ33 (Figure 5H). During the entire simulation, the interaction energy between LDHA and AZ33 remained below −100 kJ/mol. However, after 60 ns, the energy between LDHA and FX11 also dropped below −100 kJ/mol (Figure 5I), indicating the stable binding of LDHA with FX11 or AZ33.

In conclusion, our results demonstrated the robust binding between LDHA and either FX11 or AZ33.

### 3.8 In Vitro Experimental Validation

**FIGURE 6:**
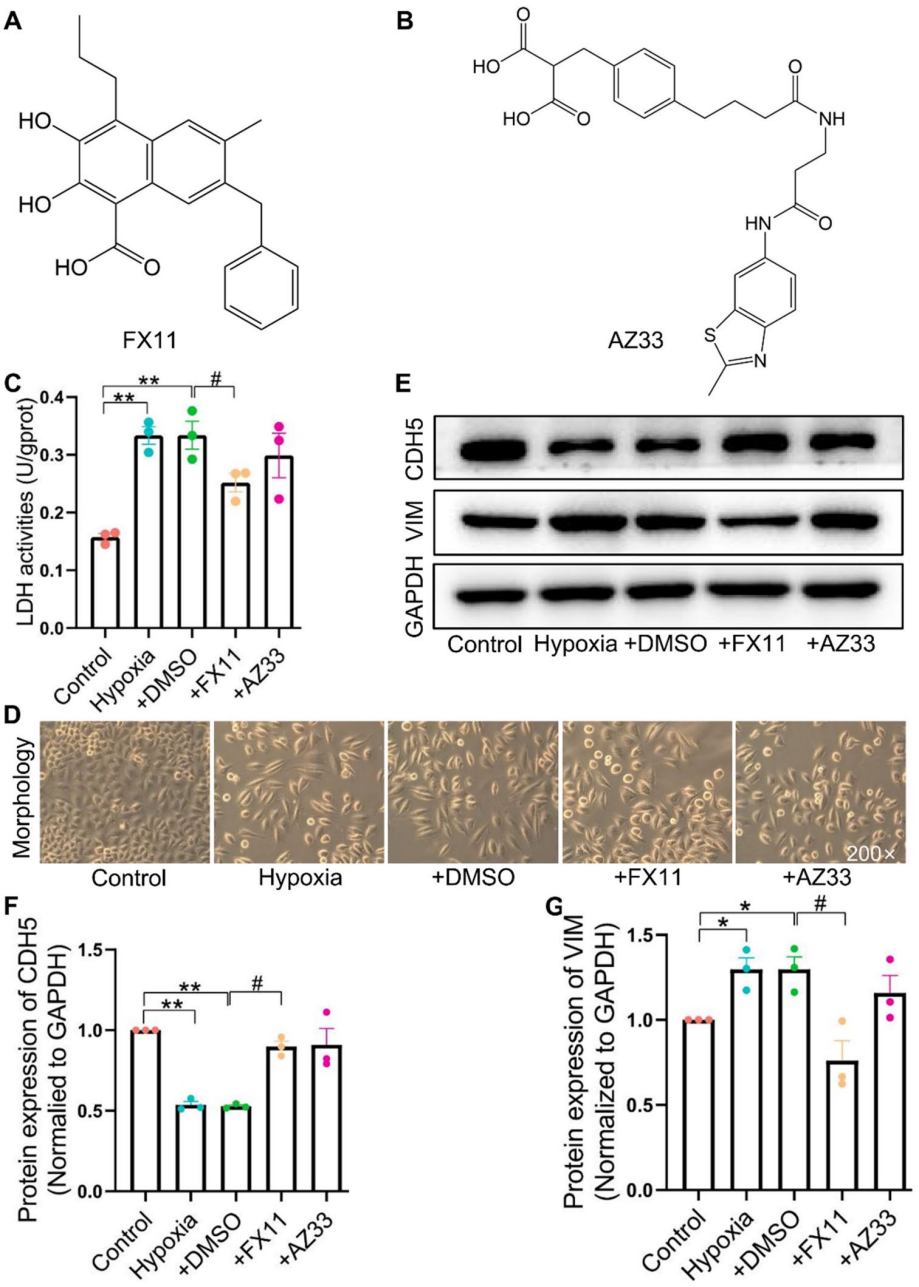
LDHA inhibition reversed hypoxia‐induced EndMT in hypoxic PAECs. Chemical structural formula of FX11 (A) and AZ33 (B). (C) LDH activity in PAECs (*n* = 3). (D) Morphology of PAECs. (E) WB. Statistical results of CDH5 (F) and VIM (G, *n* = 3). All values are expressed as mean ± S.E.M., *p < 0.05, **p < 0.01 vs. Control; #*p* < 0.05 vs. DMSO.

Given LDHA's crucial role in catalysing pyruvate to lactate, we first assessed the impact of the 2 selected compounds on LDH enzyme activity in HPAECs. The molecular structures of FX11 and AZ33 were presented in Figure 6A,B. The results showed that FX11 significantly inhibited LDH enzyme activity, while AZ33 did not exhibit a similar inhibitory function (Figure 6C). Subsequently, cell morphological experiments revealed that after exposure to hypoxia, HPAECs underwent a transition from elliptical to a spindle‐shaped morphology; notably, FX11 was able to reverse this transition, with a more favourable effect compared to AZ33 (Figure 6D). Further WB analysis revealed that, consistent with the morphological findings, FX11 significantly increased the expression of the endothelial marker CDH5 while reducing the mesenchymal marker VIM. However, AZ33 did not show a similar effect (Figure 6E–G). These findings indicated that LDHA inhibition could effectively reverse hypoxia‐induced EndMT in PAECs and FX11 was identified as a compound with the ability to inhibit EndMT.

### 3.9 In Vivo Experimental Validation

**FIGURE 7:**
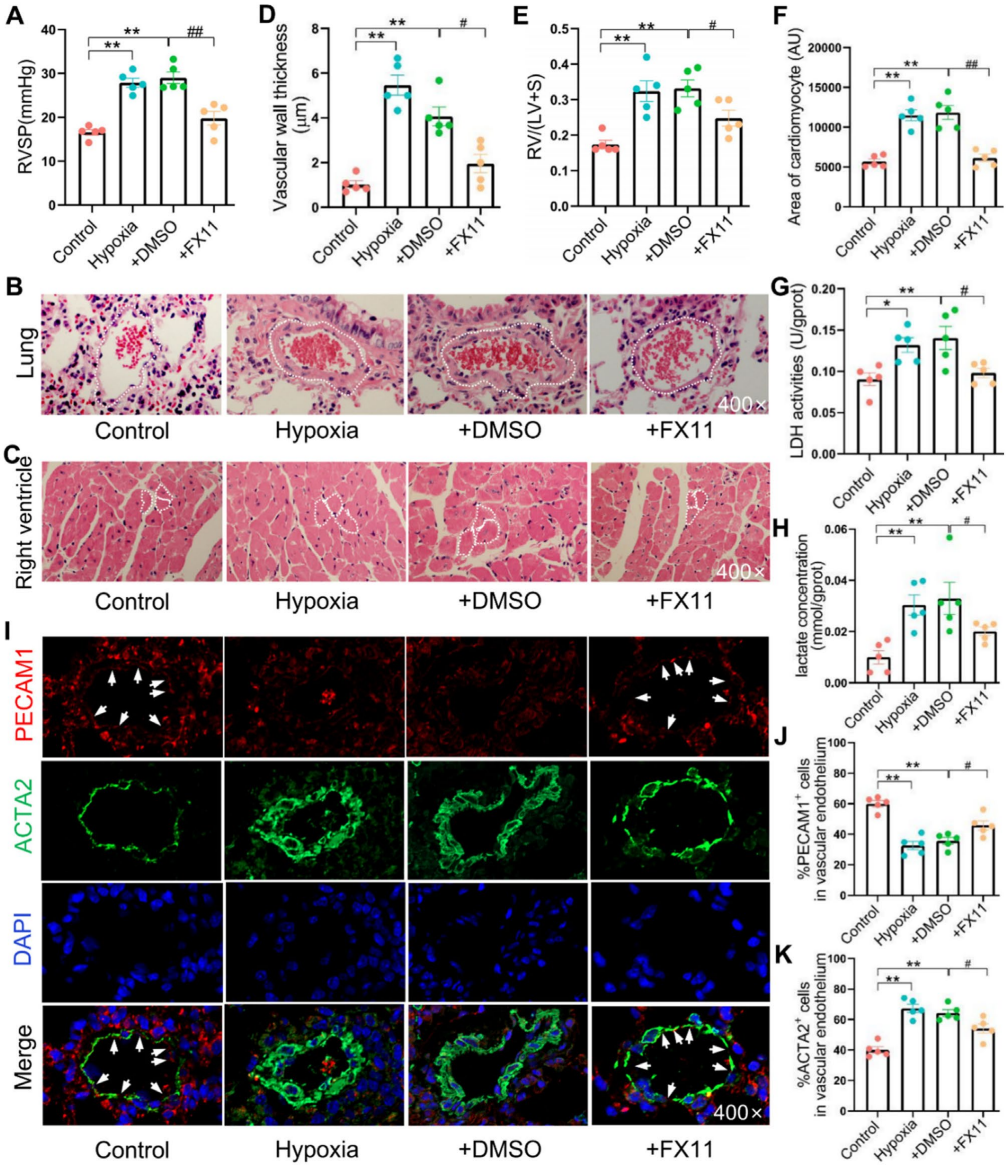
LDHA inhibition alleviated hypoxia‐induced PH through suppressing EndMT in mice. (A) RVSP. (B) HE staining of lung tissues. (C) HE staining of right ventricle tissues. (D) Vascular wall thickness. (E) RV/LV + S. (F) Area of cardiomyocyte. (G) LDH activity in lung tissues. (H) Lactate concentration in lung tissues. (I) Protein expression of PECAM1 and ACTA2 identified by IF. %PECAM1^+^ (J) and %ACTA2^+^ (K) cells in vascular endothelium. All values are expressed as mean ± S.E.M., *n* = 5, *p < 0.05, **p < 0.01 vs. Control; ^#^ *p* < 0.05, ^##^ *p* < 0.01 vs. DMSO.

To investigate the anti‐PH efficacy of LDHA inhibition, we established a PH mouse model by hypoxia exposure combined with SU5416 injection and chose FX11 as the LDHA inhibitor due to its ability to inhibit EndMT. Starting from the first day of model preparation, mice were intraperitoneally injected with the LDHA inhibitor FX11 daily until the experiment concluded. The results indicated that FX11 significantly downregulated the hypoxia‐induced increase in RVSP (Figure 7A), alleviated pulmonary artery remodelling (Figure 7B,D) and reduced right ventricular hypertrophy (Figure 7C,E,F) in PH mice. Furthermore, FX11 inhibited LDHA activity (confirmed by LDH activity and lactate concentration, Figure 7G,H) and decreased lactate concentration (Figure 7H) in lung tissue, thereby reversing EndMT (illustrated by PECAM1 and ACTA2 protein expression in pulmonary artery endothelium, Figure 7I–K). These results suggested that LDHA inhibition exerted an anti‐PH effect by repressing EndMT.

### 3.10 Mechanistic Studies

**FIGURE 8:**
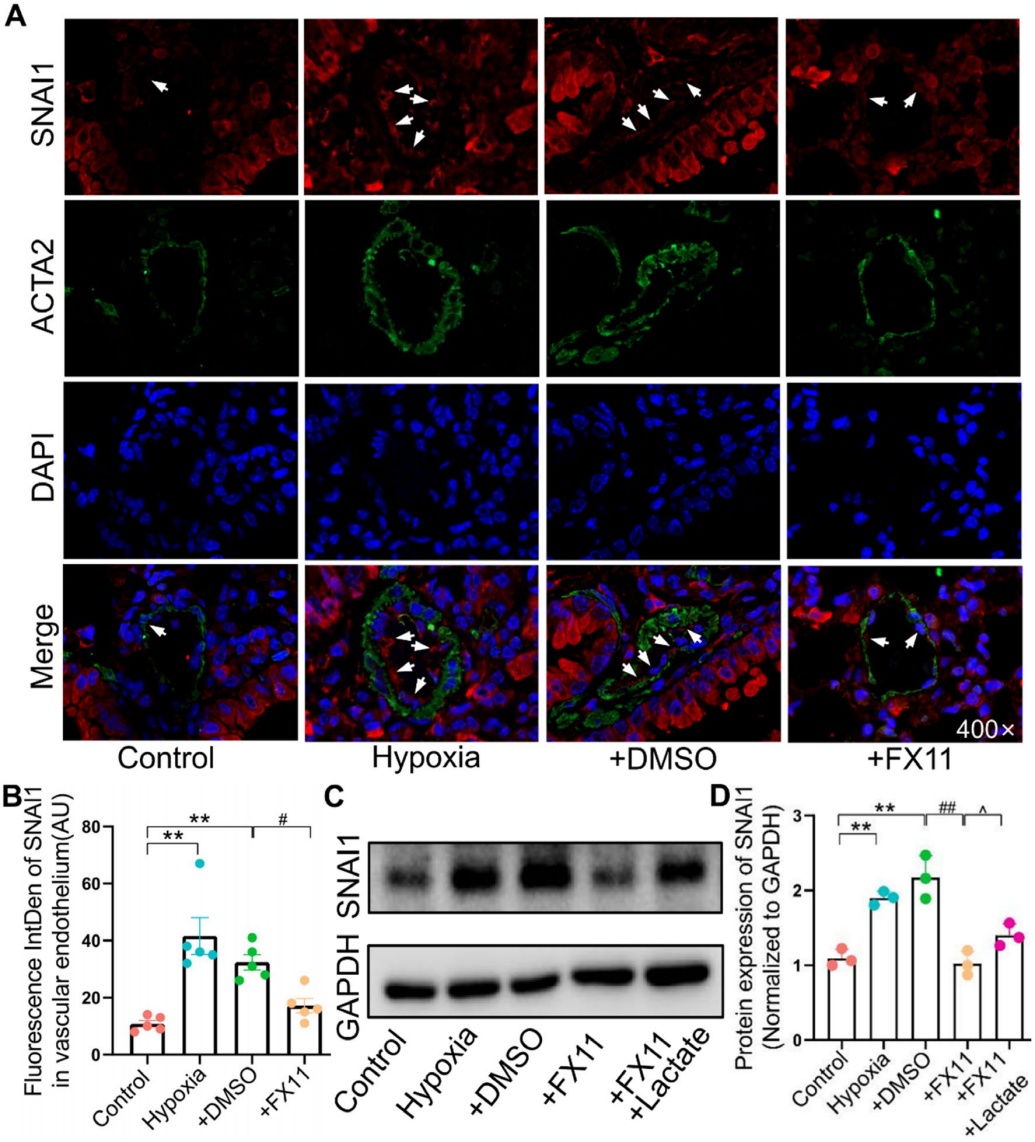
LDHA regulated EndMT via lactate‐SNAI1 axis. (A, B) SNAI1 expression in vascular endothelium detected by IF (*n* = 5). (C, D) SNAI1 protein expression in PAECs detected by WB (*n* = 3). All values are expressed as mean ± S.E.M., **p < 0.01 vs. Control; ^#^ *p* < 0.05, ^##^ *p* < 0.01 vs. DMSO; ^^^ *p* < 0.05 vs. FX11.

To elucidate the molecular mechanism underlying LDHA regulation of EndMT, we first conducted in vivo studies. The LDHA inhibitor FX11 significantly reversed the hypoxia‐induced increase in the expression of SNAI1, a key regulatory factor of EndMT, in the pulmonary artery endothelium of mice (Figure 8A,B). In vitro experiments further demonstrated that LDHA inhibition also reversed hypoxia‐induced SNAI1 upregulation in HPAECs, while lactate rescued the decrease in SNAI1 expression induced by LDHA inhibition (Figure 8C,D). Additionally, LDHA regulated lactate production in lung tissues as well (Figure 7H). Together, these findings indicated that LDHA regulated EndMT through the lactate–SNAI1 axis.

## 4 Discussion

EndMT, a crucial process in pulmonary arterial vascular remodelling, is a key initiating event in PH [29]. The pathological consequences of EndMT are diverse, involving not only the excessive generation of stromal cells such as smooth muscle cells and fibroblasts, but also the excessive deposition of extracellular matrix and increased procoagulant activity [30]. Furthermore, EndMT contributes to the deterioration of PH [31]. Given the critical role of EndMT throughout the progression of PH, the severity of the disease, and the limitations of current treatment drugs, there is an urgent need to identify new targets regulating EndMT, thus facilitating the development of novel drugs for treating PH.

In this study, 2 datasets based on hypoxic HPAECs were selected as training dataset to identify overlapping DEGs involved in EndMT. The GO analysis results indicated that the upregulated DEGs were mainly enriched in the terms about extracellular matrix and response to hypoxia. The deposition of extracellular matrix on the pulmonary arterial intima is a crucial step in pulmonary arterial remodelling [32], while the hypoxia is closely associated with the dysfunction of pulmonary artery endothelial function through the HIF‐1 signalling pathway [33], suggesting a possible close relationship between these DEGs and EndMT. According to KEGG analysis, these DEGs were found to be enriched in the HIF‐1 signalling pathway and the glycolysis/gluconeogenesis. The HIF system plays a crucial role in facilitating cellular adaptation to hypoxic stress, echoing the enrichment of “response to decreased oxygen levels” identified in the GO analysis. The HIF‐1 signalling pathway has implicated in various hypoxia‐related diseases such as cancer [34] and systemic sclerosis [35]. In the context of PH, HIF‐1 is activated by SRC overexpression, leading to the proliferation and migration of PASMCs [36]. Furthermore, it is also activated by cerebellin‐2, subsequently regulating EndMT [37]. Regarding glycolysis/gluconeogenesis, it has been identified as the primary pathway in hypoxic PASMCs, as revealed by KEGG analysis [38]. Activation of this pathway in endothelial cells has been shown to promote the development of PH [39]. Moreover, a recent study has demonstrated that HIF‐1 regulated glycolysis by targeting several glycolytic enzyme genes, including GAPDH and LDHA [40], and our results showed that there were 7 cross‐pathway DEGs present in both pathways simultaneously, indicating the potential synergistic effect of the 2 pathways. In summary, these pieces of evidence not only demonstrate the consistency of the results from the 2 different enrichment analysis methods but also underscore the critical roles of the 2 signalling pathways enriched through KEGG analysis in the progression of EndMT.

To identify core target in EndMT, we initially employed the MCC algorithm in Cytoscape to pinpoint 5 hub genes from the DEGs. Notably, the analysis, using cross‐pathway DEGs and ClueGo, demonstrated that these hub genes were closely associated with the HIF‐1 signalling pathway and glycolysis/gluconeogenesis enriched by KEGG. This indicated that, in this study, the enrichment results derived from the KEGG analysis were in line with those obtained through the MCC algorithm, not only highlighting the credibility of the used datasets and the results of this bioinformatics analysis, but also emphasising the efficacy of combined application of KEGG and the MCC algorithm in screening for hub genes from bioinformatics datasets. LDHA was then identified as a key gene in EndMT among the 5 hub genes for 3 reasons. Firstly, it ranked second in the MCC algorithm results. Secondly, it was the only significantly upregulated hub gene in the validation set GSE113439 from clinical samples. Thirdly, it showed the strongest correlation with EndMT marker genes in the validation set. The subsequent GSEA analysis result indicated that the key gene LDHA was enriched in 2 signalling pathways mentioned above. Since both pathways play crucial roles in EndMT, these results underscored the reliability of LDHA as a key gene in this process.

Lactate dehydrogenase primarily consists of two subunits: LDHA and LDHB. LDHB has a higher affinity for lactate, catalysing the conversion of lactate to pyruvate [41]; LDHA is considered a crucial enzyme in the conversion of pyruvate to lactate [42]. LDHA exhibits upregulated expression under conditions such as hypoxia [43], which has been confirmed in hypoxic pulmonary arterial endothelium and PAECs by our results. Additionally, this upregulation results in increased lactate production owing to elevated enzyme activity [44], which was also validated by data from our clinical and animal sample. The accumulated lactate is then transported out of the cells by monocarboxylate transporters [45]. Subsequently, it activates G‐protein‐coupled receptor 81 to regulate immune reaction [46] or induce lactylation of target proteins to modulate pathophysiological processes [47, 48]. In view of its vital role in lactate production and the significance of lactate in multiple pathophysiological processes, LDHA has been recognised as a therapeutic target in various types of cancer [45, 47, 49, 50]. In silico [38] and experimental [51] research have suggested the roles of LDHA in the proliferation of PASMCs in PH. Importantly, the HIF‐1 signalling pathway, enriched by KEGG, ClueGo, and GSEA in this study, not only regulates the transcription of LDHA [52], but also modulates hypoxia‐induced EndMT in PH [37]. These evidences support our bioinformatics analysis and indicate that LDHA might be a therapeutic target in EndMT during PH.

Then, FX11 and AZ33 were selected as LDHA inhibitors through database screening, molecular docking, and MDS to verify the potential of LDHA as a therapeutic target for PH. Compared to AZ33, FX11 exhibited more pronounced anti‐EndMT effects in hypoxic HPAECs. FX11 is a selective small‐molecule LDHA inhibitor that acts competitively with NADH to bind to the LDHA protein, resulting in the inhibition of LDHA activity [53]. This mechanism has been shown to exhibit significant anticancer effects [54, 55]. Consistently, our in vivo results indicated that the inhibition of LDHA activity mediated by FX11 reversed hypoxia‐induced PH via suppressing EndMT, suggesting the role of LDHA inhibition as an effective option for PH treatment. Interestingly, the mechanism of action of the drug Nedosiran, recently approved by the U.S. Food and Drug Administration for the treatment of primary hyperoxaluria type 1, acts by directly interfering with LDHA mRNA transcription [56], providing further promising evidence for the potential of LDHA as a therapeutic target for PH.

SNAI1 is a crucial member of the SNAIL family. The four C2H2 zinc finger domains located at its carboxyl terminus serve as DNA‐binding sites, enabling it to bind to the E‐box in the promoter region of target genes and thereby regulate transcription [57]. As a transcription factor, SNAI1 can inhibit E‐cadherin while activating N‐cadherin, thereby triggering epithelial‐mesenchymal transition (EMT) [58]. Our results have indicated that LDHA inhibition reversed the hypoxia‐induced increase in CDH5 (VE‐cadherin) expression. Considering this finding, it is plausible that this reversal is regulated by SNAI1‐mediated transcription of CDH5. In this study, LDHA was found to promote lactate production, and lactate restored the decrease in SNAI1 expression caused by LDHA inhibition. Furthermore, SNAI1 has been identified as a key regulatory factor for EndMT in PH [59]. Consequently, we propose that the upregulated lactate‐SNAI1 axis is involved in EndMT, functioning as a downstream signalling pathway of LDHA. Since lactylation of SNAI1 protein has been shown to regulate EndMT after myocardial infarction [60], and lactylation modification enhances protein stability by inhibiting the ubiquitin‐proteasome system [61], this implies that the hypoxia‐mediated increase of SNAI1 expression in PAECs may be associated with enhanced protein lactylation.

However, this study has several limitations: [1] The specific lysine residue(s) on the SNAI1 protein that undergo lactylation under hypoxic PH conditions remain(s) unclear; the enzyme(s) responsible for catalysing the lactylation of lysine residues are also unknown; [2] Further confirmation is needed regarding whether the ubiquitin‐proteasome system, which acts on the SNAI1 protein, is inhibited by lactylation; [3] We failed to validate the expression of LDHA and SNAI1 in pulmonary artery endothelium using lung tissues obtained from clinical samples. Hence, further in‐depth research will be instrumental in overcoming these limitations.

## 5 Conclusion

We identified 310 overlapping DEGs that were upregulated and 229 that were downregulated from 2 datasets of hypoxia‐induced EndMT cell models. These upregulated DEGs were primarily enriched in the HIF‐1 signalling pathway and glycolysis/gluconeogenesis. By employing a combination of KEGG analysis and MCC algorithms, we pinpointed 5 hub genes and revealed that these hub genes were enriched in these 2 pathways. Through an analysis of expression and correlation in a validation set, LDHA emerged as the key gene among the hub genes. Its enrichment, protein expression, and enzyme activity were further corroborated by in silico analysis and experimental testing. Subsequently, experimental results revealed that compound FX11, one of 4 LDHA inhibitors screened from 2 online databases, was able to reverse hypoxia‐induced PH by inhibiting EndMT through suppression of the lactate‐SNAI1 axis. These discoveries offer an effective method for identifying hub genes in bioinformatic analysis and suggest LDHA as a potential therapeutic target for PH treatment.

## Author Contributions

**Maozhong Yao:** formal analysis (equal), funding acquisition (equal), software (lead), visualization (lead), writing – original draft (lead). **Keyan Zhong:** investigation (equal), validation (equal). **Xinbin Zheng:** investigation (equal), validation (equal). **Zhaoxin Yang:** formal analysis (equal). **Chunying Li:** conceptualization (equal), resources (equal). **Yong Gu:** funding acquisition (equal), resources (equal), supervision (lead), writing – review and editing (equal). **Zhanjuan Chen:** conceptualization (equal), funding acquisition (equal), methodology (lead), project administration (lead), writing – review and editing (equal).

## Ethics Statement

All human sample collections were approved by the Ethics Committee of Hainan Hospital, Guangdong Provincial Hospital of Chinese Medicine (No. HNSZYY‐2024‐LL‐071). Animal care and experimental procedures were conducted following a protocol approved by the Institutional Animal Care and Use Committee of Hainan Hospital, Guangdong Provincial Hospital of Chinese Medicine (No. IACUC‐HHGPHCM‐2404002).

## Conflicts of Interest

The authors declare no conflicts of interest.

## Supporting information


Appendix S1.

## Data Availability

The data that support the findings of this study are available from the corresponding author upon reasonable request.
