# Supplementary material for: Identification of LDHA as a Potential Therapeutic Target for Pulmonary Hypertension Through Modulation of Endothelial‐To‐Mesenchymal Transition
Source: J Cell Mol Med. 2025 Jul 8;29(13):e70692. doi: 10.1111/jcmm.70692 (PMC12237618; doi:10.1111/jcmm.70692)
Supplement: Supplementary file 1 — Appendix S1. [file JCMM-29-e70692-s001.docx]

Supplementary Table S1. LDHA inhibitors screened by BindingDB

| No. | Compound ID In BindingDB | Compound InChIKey |
| --- | --- | --- |
| 1 | BDBM86116 | WKUSZUMHMJWPPR-UHFFFAOYSA-N |
| 2 | BDBM86121 | DNTIJCDTDDGTKN-UHFFFAOYSA-N |
| 3 | BDBM86122 | WXPAWXCZGXNAFZ-UHFFFAOYSA-N |
| 4 | BDBM86123 | MTRGAKUTJCSVKA-UHFFFAOYSA-N |
| 5 | BDBM86124 | GZMFZHIPGZSUHI-UHFFFAOYSA-N |
| 6 | BDBM86125 | NQKOQSKMBSAXTD-UHFFFAOYSA-N |
| 7 | BDBM86126 | OVXHZNUBLPBIEB-UHFFFAOYSA-N |
| 8 | BDBM86130 | YPEMYPUMNYOIMU-UHFFFAOYSA-N |
| 9 | BDBM86131 | ULZFONRJONTZBF-UHFFFAOYSA-N |
| 10 | BDBM86132 | FVQKLUULQXPDHM-UHFFFAOYSA-N |
| 11 | BDBM86133 | GADNEHRFYQNKCC-UHFFFAOYSA-N |
| 12 | BDBM86134 | FKUWEZQWIUGSHV-UHFFFAOYSA-N |
| 13 | BDBM86135 | XAQKXAUMONXVAC-UHFFFAOYSA-N |
| 14 | BDBM86136 | OVKDDZVXJKGLNM-UHFFFAOYSA-N |
| 15 | BDBM86137 | SGFJAJFBGVAOFW-UHFFFAOYSA-N |
| 16 | BDBM86117 | YPYAGNMJHWIZMQ-UHFFFAOYSA-N |
| 17 | BDBM86118 | WOIJKOZDDUWHBG-UHFFFAOYSA-N |
| 18 | BDBM86119 | PSUIIKIEUATWCZ-UHFFFAOYSA-N |
| 19 | BDBM86120 | XLYNOVXELOHZDY-UHFFFAOYSA-N |
| 20 | BDBM86127 | BVTLJYZGVOIQHN-UHFFFAOYSA-N |
| 21 | BDBM86128 | FBTWWPKAGBUCIB-UHFFFAOYSA-N |
| 22 | BDBM86129 | ZBBWIKBLGUCZOQ-UHFFFAOYSA-N |
| 23 | BDBM86138 | DHKHILIBZATWFN-UHFFFAOYSA-N |
| 24 | BDBM86113 | XQHMUSRSLNRVGA-KBIXCLLPSA-L |
| 25 | BDBM86114 | TXDOUQMFGDSHDX-ANJHZYCWSA-L |
| 26 | BDBM86115 | LQAKRSXPUIKTEV-UHFFFAOYSA-N |
| 27 | BDBM23251 | FVZITYNLUYJDOE-UHFFFAOYSA-N |
| 28 | BDBM50339607 | KGXDBNBXAPVUIM-UHFFFAOYSA-N |
| 29 | BDBM50066974 | LVPYVYFMCKYFCZ-UHFFFAOYSA-N |
| 30 | BDBM50219487 | SJJCQDRGABAVBB-UHFFFAOYSA-N |
| 31 | BDBM50078827 | BOUOQESVDURNSB-UHFFFAOYSA-N |
| 32 | BDBM50192453 | UDMBCSSLTHHNCD-KQYNXXCUSA-L |

Supplementary Table S2. LDHA inhibitors screened by ZINC20

| No. | Compound ID  in ZINC20 | Compound InChIKey |
| --- | --- | --- |
| 1 | ZINC000098210307 | RZBCPMYJIARMGV-UHFFFAOYSA-N |
| 2 | ZINC000299819923 | GLDDJXYFHWRGPI-ILBGXUMGSA-N |
| 3 | ZINC000299819927 | GLDDJXYFHWRGPI-NOZRDPDXSA-N |
| 4 | ZINC000299819934 | GLDDJXYFHWRGPI-UKILVPOCSA-N |
| 5 | ZINC000299819940 | GLDDJXYFHWRGPI-ZCYQVOJMSA-N |
| 6 | ZINC000084690296 | SGFJAJFBGVAOFW-UHFFFAOYSA-N |
| 7 | ZINC000013435952 | QYDDTEVBIYQMGD-UHFFFAOYSA-N |
| 8 | ZINC000013435956 | LVPYVYFMCKYFCZ-UHFFFAOYSA-N |
| 9 | ZINC000031773899 | AHYCTVUVTZKPOE-UHFFFAOYSA-N |
| 10 | ZINC000063796290 | KGXDBNBXAPVUIM-UHFFFAOYSA-N |
| 11 | ZINC000146883754 | YPPFWRWCZNXINO-UHFFFAOYSA-N |
| 12 | ZINC000002758482 | DLDDCGAZVYQHHN-SFHVURJKSA-N |
| 13 | ZINC000002758483 | DLDDCGAZVYQHHN-GOSISDBHSA-N |
| 14 | ZINC000006523896 | FVZITYNLUYJDOE-UHFFFAOYSA-N |


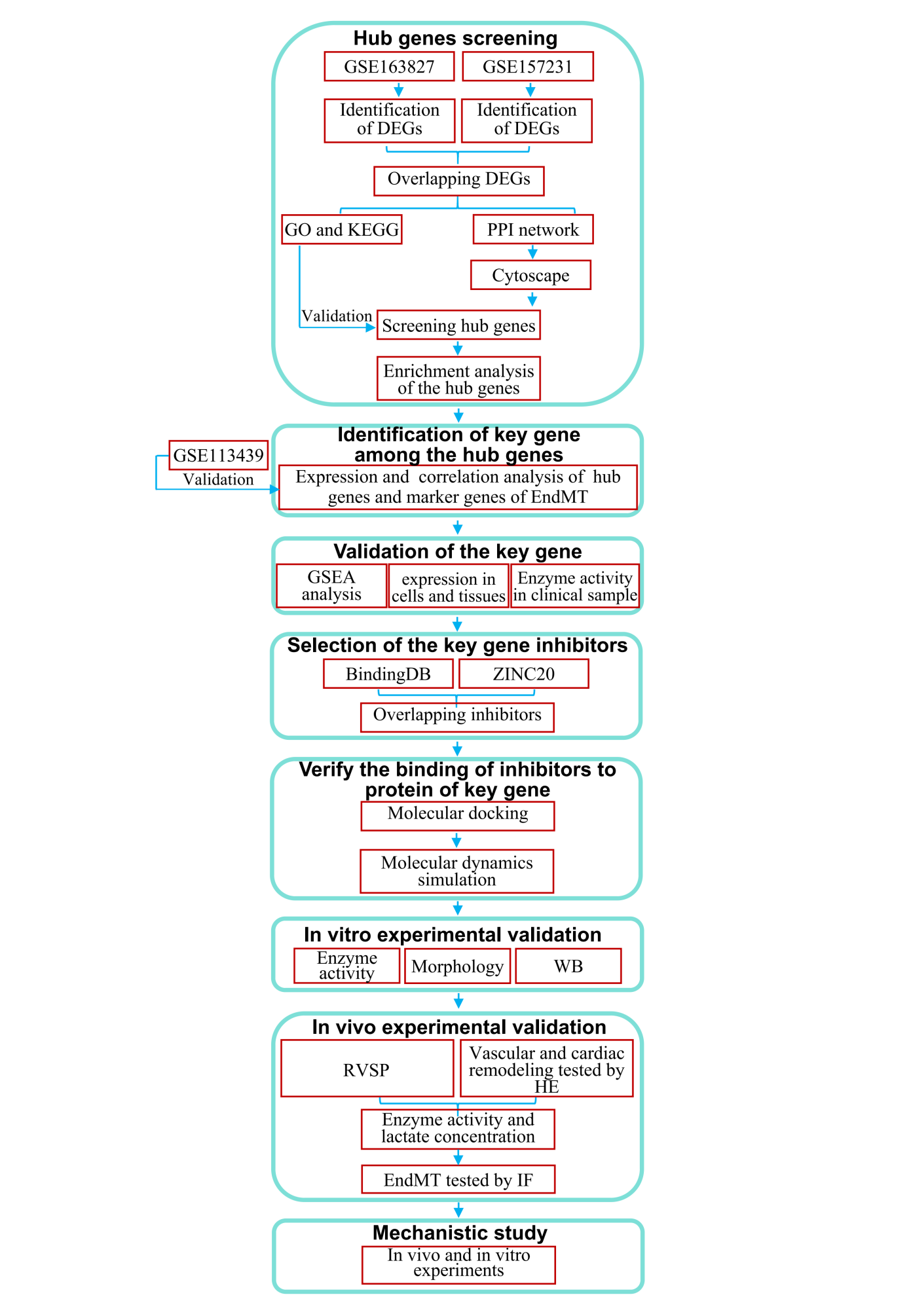


Supplementary FIGURE S1. The flowchart for the study.


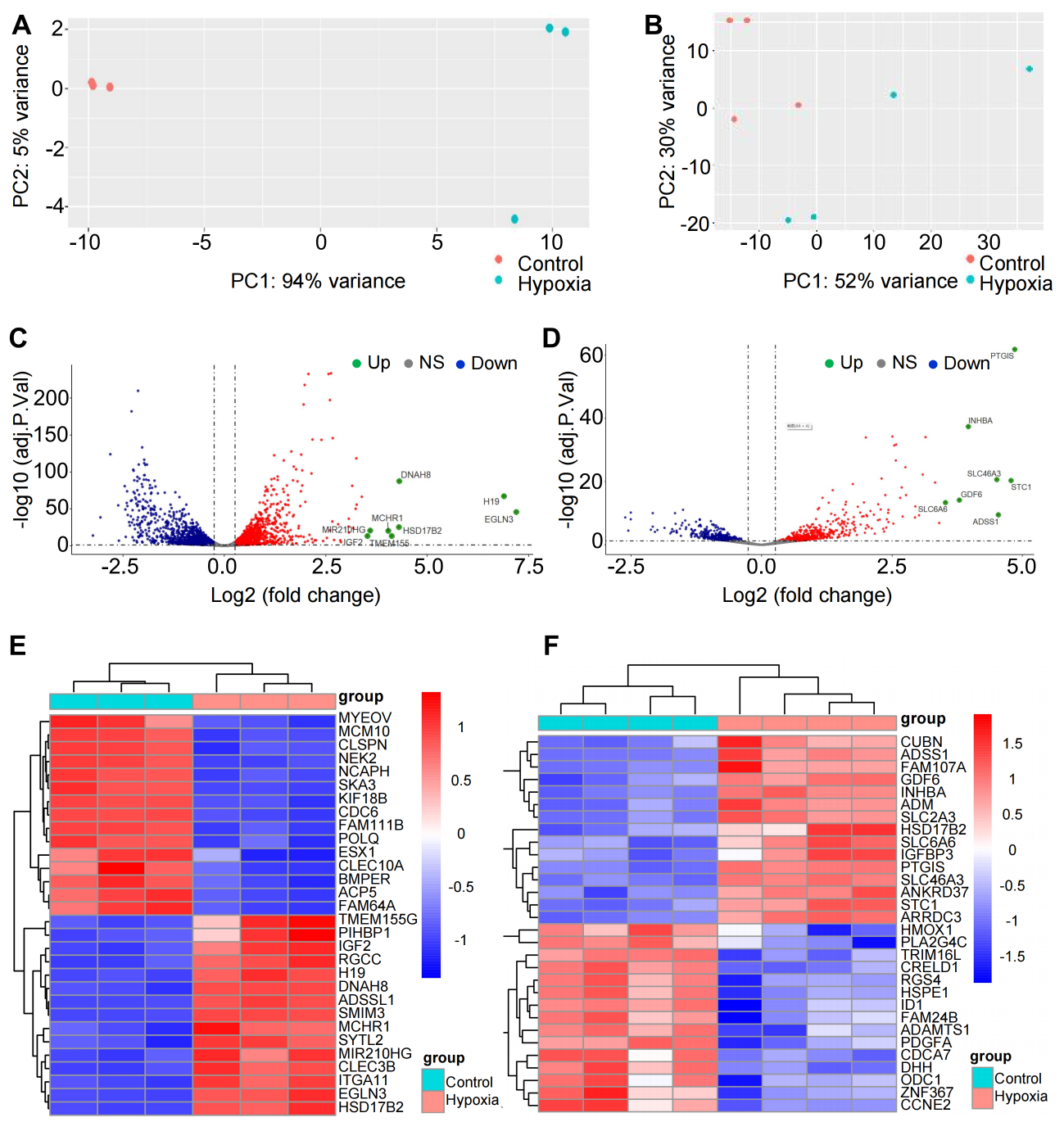


Supplementary FIGURE S2. Identification of DEGs. PCA for GSE163827 **(A)** and GSE157231 **(B)**. Volcano plots of GSE163827 **(C)** and GSE157231 **(D)**. Heatmaps of GSE163827 **(E)** and GSE157231 **(F)**.


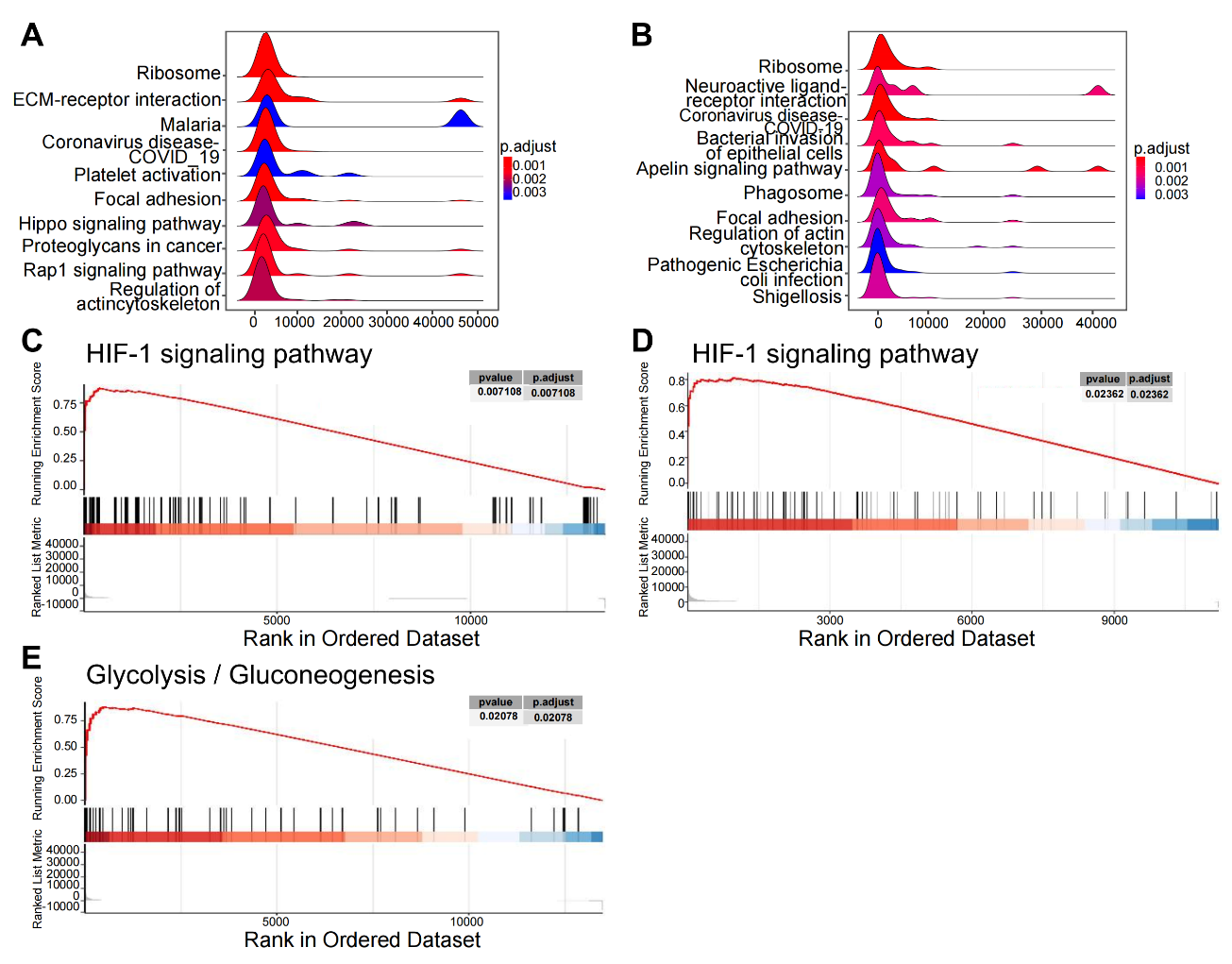


Supplementary FIGURE S3. GSEA between the high LDHA expression group and low LDHA expression group. Ridge plot of GSE163827 **(A)** and GSE157231 **(B)**. HIF-1 signaling pathway **(C)** and Glycolysis/Gluconeogenesis **(E)** in GSE163827. **(D)** HIF-1 signaling pathway in GSE157231.
